# Declining racial and ethnic representation in clinical academic medicine: A longitudinal study of 16 US medical specialties

**DOI:** 10.1371/journal.pone.0207274

**Published:** 2018-11-16

**Authors:** Elle Lett, Whitney U. Orji, Ronnie Sebro

**Affiliations:** 1 Perelman School of Medicine, University of Pennsylvania, Philadelphia, Pennsylvania, United States of America; 2 Department of Radiology, University of Pennsylvania, Philadelphia, Pennsylvania, United States of America; 3 Department of Biostatistics, Epidemiology, and Informatics, University of Pennsylvania, Philadelphia, Pennsylvania, United States of America; 4 Department of Genetics, University of Pennsylvania, Philadelphia, Pennsylvania, United States of America; Indiana University, UNITED STATES

## Abstract

**Objective:**

To evaluate trends in racial, ethnic, and sex representation at US medical schools across 16 specialties: internal medicine, pediatrics, surgery, psychiatry, radiology, anesthesiology, obstetrics and gynecology, neurology, family practice, pathology, emergency medicine, orthopedic surgery, ophthalmology, otolaryngology, physical medicine and rehabilitation, and dermatology. Using a novel, Census-derived statistical measure of diversity, the S-score, we quantified the degree of underrepresentation for racial minority groups and female faculty by rank for assistant, associate, and full professors from 1990–2016.

**Methods:**

This longitudinal study of faculty diversity uses data obtained from the American Association of Medical Colleges (AAMC) Faculty Roster from US allopathic medical schools. The proportion of professors of racial minority groups and female faculty by rank was compared to the US population based on data from the US Census Bureau. The Roster includes data on 52,939 clinical medical faculty in 1990, and 129,545 in 2016, at the assistant professor level or higher.

The primary measure used in this study was the S-score, a measure of representation based on the probability of the observed frequency of faculty from a racial/ethnic group and sex, given the racial and ethnic distribution of the US. Pearson correlations and 95% confidence intervals for S-score with time were used to measure trends.

**Results:**

Blacks and Hispanics showed statistically significant trends (p<0.05) towards increasing underrepresentation in most specialties and are more underrepresented in 2016 than in 1990 across all ranks and specialties analyzed, except for Black females in obstetrics & gynecology. White females were also underrepresented in many specialties and in a subset of specialties trended toward greater underrepresentation.

**Conclusions:**

Current efforts to improve faculty diversity are inadequate in generating an academic physician workforce that represents the diversity of the US. More aggressive measures for faculty recruitment, retention, and promotion are necessary to reach equity in academia and healthcare.

## Introduction

The importance of diversity in the medical workforce in the US is well-established. Several studies have shown that Black and Hispanic physicians are more likely to care for underserved populations, including racial minorities and uninsured patients[1,2]. Physician diversity has also been linked to better patient outcomes in primary care[3]. Further, a more diverse faculty increases the ability of institutions to train physicians to practice in racially heterogenous populations, and promotes biomedical research that addresses disparities in health access/outcomes in marginalized communities[4]. These results suggest that improving racial and ethnic minority representation among physicians may be integral to reducing health disparities and achieving health equity [5]. The Diversity 3.0 initiative proposed in 2010 by the American Academy of Medical Colleges (AAMC) stresses that diversity and inclusion in academic medicine are important for addressing health disparities in the US[6].

There is currently no standardized metric for evaluating representation in medicine. In an effort to investigate trends in their respective fields, scientists have analyzed the number and percent/proportions of physicians at the resident and faculty level across multiple specialties and medicine in the US overall[7–15]. These studies report modest gains in the number and/or percent of racial minorities and females. However, as our study shows, inference based on evaluation of these metrics often lead to erroneous conclusions regarding diversity. Many of these studies make note of the increasing diversity of the US population but none statistically quantify the discrepancy between representation in medicine and the general population, in keeping with the AAMC definition of under-represented in medicine (URM)[16]. The two primary metrics used to assess URM diversity in the literature have been the (1) change in the number and (2) change in the proportion/percentages of URM[7–15]. The change in the number is informative but does not take into consideration the concomitant change in the size of the faculty and does not consider the concomitant changes in the US Census. For example, a university with 100 URM faculty can state that their URM faculty increased by 10% over 5 years from 100 URM faculty to 110 URM faculty, however, if simultaneously the total faculty body increased from 2000 faculty to 2400 faculty over the same time period of 5 years, then this “increase” is actually a relative decrease from 100/2000 (5%) to 110/2400 (4.58%). Here, we propose a Census-derived metric that measures racial and sex diversity in the context of the of the US population. This statistic is novel and has not been used previously in the literature. As described above and shown in S1 Appendix, other commonly used metrics do not accurately measure change in diversity, so we directly compared the demographics of the academic physician workforce with the demographics of the US and quantified changes in the severity of underrepresentation for each race/ethnicity and sex between 1990 and 2016. We show that this method can be used to objectively measure underrepresentation in academic medicine.

## Methods

### Faculty data

Publicly available data from the AAMC Faculty Administrative Management Online User System (FAMOUS) Faculty Roster from 1990–2016 were utilized[17]. These data can be obtained from the AAMC through online data request. These data included aggregate counts of faculty by rank, specialty, sex, and race/ethnicity voluntarily reported by US allopathic medical schools. We analyzed trends for clinical medical faculty at the assistant, associate, and full professor level in 16 clinical specialties: internal medicine, pediatrics, surgery, psychiatry, radiology, anesthesiology, obstetrics and gynecology (OB/GYN), neurology, family practice, pathology, emergency medicine, orthopedic surgery, ophthalmology, otolaryngology, physical medicine and rehabilitation (PMR), and dermatology. Faculty racial groups were self-reported as Asian, American Indian or Alaska Native (AIAN), Hispanic, Latino, or of Spanish Origin (Hispanic), African-American or Black (Black), Unknown, Multiple Race–Hispanic, and Multiple Race–Non-Hispanic (MRNH). The counts for the categories, Hispanic, Latino, or of Spanish Origin, and Multiple Race—Hispanic, were combined and compared to Hispanics according to the Census data.

### Census data

The proportions of American Indian/Alaska Native (AIAN), Asian, Black/African-American, Hispanic, Native Hawaiian/Other Pacific Islander (NHOPI) and White non-Hispanic individuals from 1990–2016 were obtained from data from the decennial US Census and the American Community Survey [18–20]. These data can be obtained from census.gov (for the 1990 and 2000 Census), and factfinder.census.gov (for the 2010 Census and 2011–2016 ACS). AIAN and NHOPI groups were excluded from this analysis due to incomplete reporting (data on NHOPI were not available to prior 2005), and sparse frequencies across specialties. We restrict the calculations to the US population between the ages of 30–84 to represent individuals who are of the age to have completed training and be actively practicing medicine as faculty. Linear regression models were used to interpolate the proportions of each race/ethnicity for years when data was not available. These proportions can be found in Table 1.

**Table 1:** US population estimates by race/ethnicity and sex based on Census data.

Year	Asian		Black		Hispanic		White	
	Male	Female	Male	Female	Male	Female	Male	Female
1990	0.01	0.01	0.05	0.06	0.03	0.03	0.38	0.42
1991	0.01	0.01	0.05	0.06	0.03	0.04	0.38	0.42
1992	0.01	0.02	0.05	0.06	0.04	0.04	0.38	0.41
1993	0.01	0.02	0.05	0.06	0.04	0.04	0.38	0.41
1994	0.01	0.02	0.05	0.06	0.04	0.04	0.38	0.41
1995	0.01	0.02	0.05	0.06	0.04	0.04	0.37	0.41
1996	0.02	0.02	0.05	0.06	0.04	0.04	0.37	0.40
1997	0.02	0.02	0.05	0.06	0.04	0.04	0.37	0.40
1998	0.02	0.02	0.05	0.06	0.04	0.04	0.37	0.40
1999	0.02	0.02	0.05	0.06	0.05	0.05	0.37	0.40
2000	0.02	0.02	0.05	0.06	0.05	0.05	0.37	0.39
2001	0.02	0.02	0.05	0.06	0.05	0.05	0.36	0.39
2002	0.02	0.02	0.05	0.06	0.05	0.05	0.36	0.39
2003	0.02	0.02	0.05	0.06	0.05	0.05	0.36	0.38
2004	0.02	0.02	0.05	0.06	0.05	0.05	0.35	0.38
2005	0.02	0.02	0.05	0.06	0.06	0.06	0.35	0.37
2006	0.02	0.02	0.05	0.06	0.06	0.06	0.35	0.37
2007	0.02	0.03	0.05	0.06	0.06	0.06	0.35	0.37
2008	0.02	0.03	0.05	0.06	0.06	0.06	0.34	0.36
2009	0.02	0.03	0.05	0.06	0.06	0.06	0.34	0.36
2010	0.02	0.03	0.05	0.06	0.06	0.06	0.34	0.35
2011	0.02	0.03	0.05	0.06	0.07	0.07	0.33	0.35
2012	0.03	0.03	0.05	0.06	0.07	0.07	0.33	0.35
2013	0.03	0.03	0.05	0.06	0.07	0.07	0.33	0.35
2014	0.03	0.03	0.05	0.06	0.07	0.07	0.33	0.34
2015	0.03	0.03	0.05	0.06	0.07	0.07	0.33	0.34
2016	0.03	0.03	0.05	0.06	0.07	0.07	0.32	0.34

### Statistical analyses

We defined the S-score based on the binomial probability of observing as extreme or more extreme (smaller) proportion of the race, ethnicity, and sex combination in question for a specified specialty and rank, assuming the null hypothesis is correct. The null hypothesis was based on the AAMC definition of underrepresented in medicine (URM)[16], that the proportion of each race/ethnicity, should be equal to the proportion of that same race/ethnicity in the US general population based on US Census data. The S-score is as follows:

S−score=−log10(∑i=0k(Ntotali)pcensusi(1−pcensus)Ntotal−i)

where k is the number of individuals belonging to the race/ethnicity and sex in question for a given specialty and rank, p_Census_ is the proportion of the same race/ethnicity and sex based on US Census data, N_total_ is the total number of faculty in that specialty at the same rank. The S-score is therefore the negative log base 10 of the cumulative binomial probability of observing k or fewer individuals belonging to the race/ethnicity in question assuming that the study population is a random sample from the US general population. Therefore, the S-score measures the deviation of the proportion of individuals from a race/ethnicity and sex from that group’s proportion in the US population. This is exactly equivalent to the commonly used statistical test of measuring the deviation of a sample proportion from a known population proportion, where the sample is the academic medical faculty, and the population is the census. In this way, we can directly measure how severely the US medical faculty underrepresents minority groups in the US population they serve. An S-score of 300 corresponds to a probability of 1x10^-300^ of observing the given proportion or fewer in the faculty assuming that the faculty are a random sample from the general US population. Higher S-scores indicate more severe underrepresentation. Here we define underrepresentation as an S-score greater than 1.602, indicating a p-value of less than 0.025, and overrepresentation as an S-score less than 0.0109, indicating a p-value greater than 0.975. Other thresholds can be applied but we chose these criteria based on the commonly accepted p-value of 0.05, so that only 5% of the population would either be over- or under-represented. S-scores vary from 0 to infinity, although the upper limit calculated in practice is typically restricted based on the statistical software utilized. A detailed explanation of the motivation for the S-score, and its advantages over previous methods of tracking changes in diversity, is provided in S1 Appendix.

For the eight specialties with the greatest number of faculty at the assistant professor level or higher in 2016 (internal medicine, pediatrics, surgery, psychiatry, radiology, anesthesiology, obstetrics and gynecology, and neurology), we graphically analyzed trends in representation using S-scores for Asian, Black, Hispanic White males and females from 1990–2016 and compared to raw counts and percentages of the same groups. For all specialties, we used Pearson correlation coefficients and their corresponding 95% confidence intervals (CIs) to describe the trend for representation of each race/ethnicity and sex group. A positive correlation indicates a trend toward increased underrepresentation, and a negative correlation indicates a trend toward representation that approaches parity with the US population. CIs are reported rather than p-values because they are preferred according to the SAMPL Guidelines for statistical reporting in biomedical research[21]. CIs that do not span 0 indicate statistically significant associations at the type 1 error rate (α) of 0.05 (p-value <0.05). All analyses were conducted in the R statistical package, version 3.3.3[22]. This study was approved by the University of Pennsylvania institutional review board (IRB), and the need for signed inform consent was waived.

## Results

As described in the Methods, this analysis utilized FAMOUS Faculty Roster data from the 147 US allopathic medical schools. This included 52,939 clinical faculty in 1990 and 129,545 clinical faculty in 2016.

### Assistant professors

**Fig 1:**
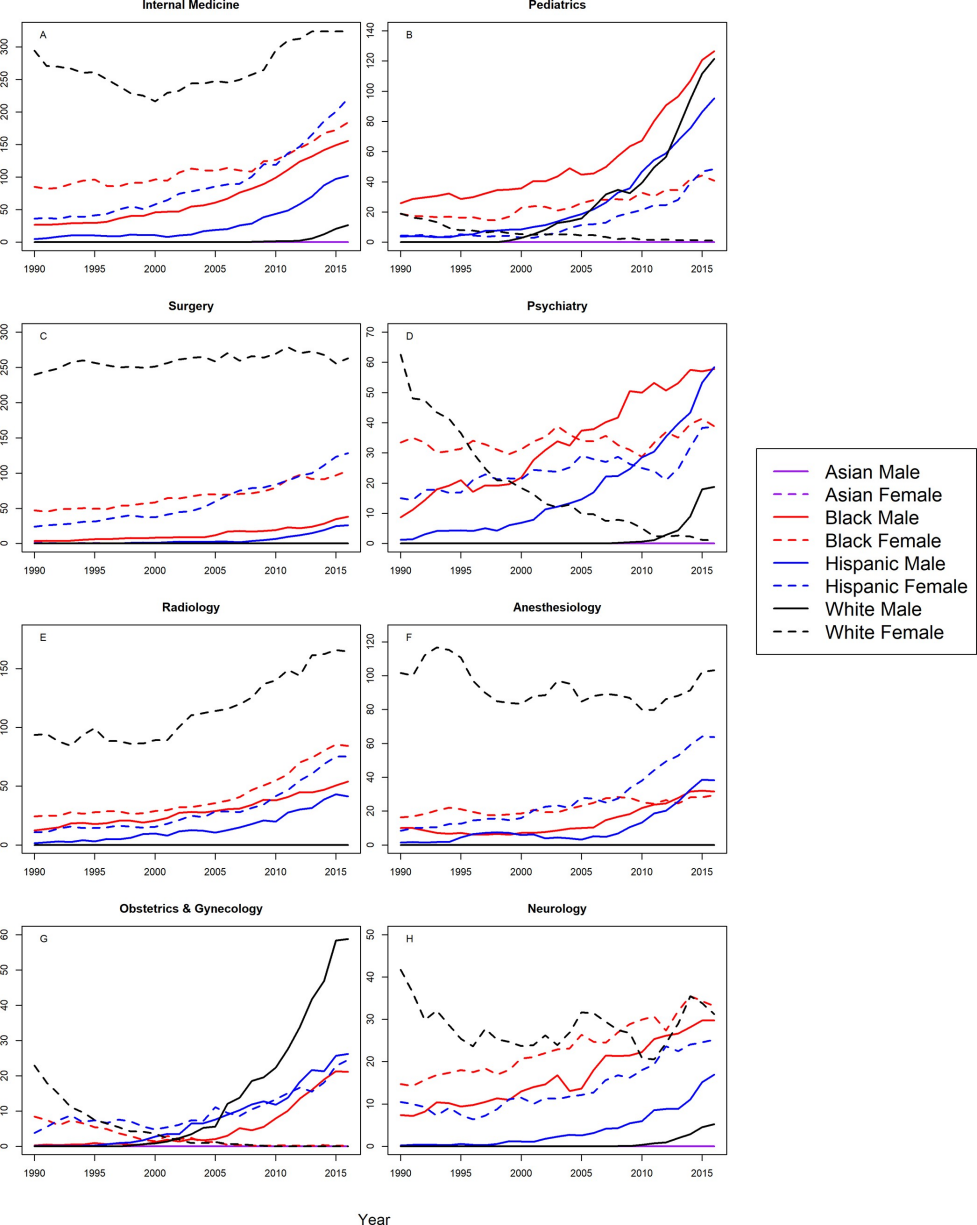
S-score for assistant professors by sex, race/ethnicity and department.

**Fig 2:**
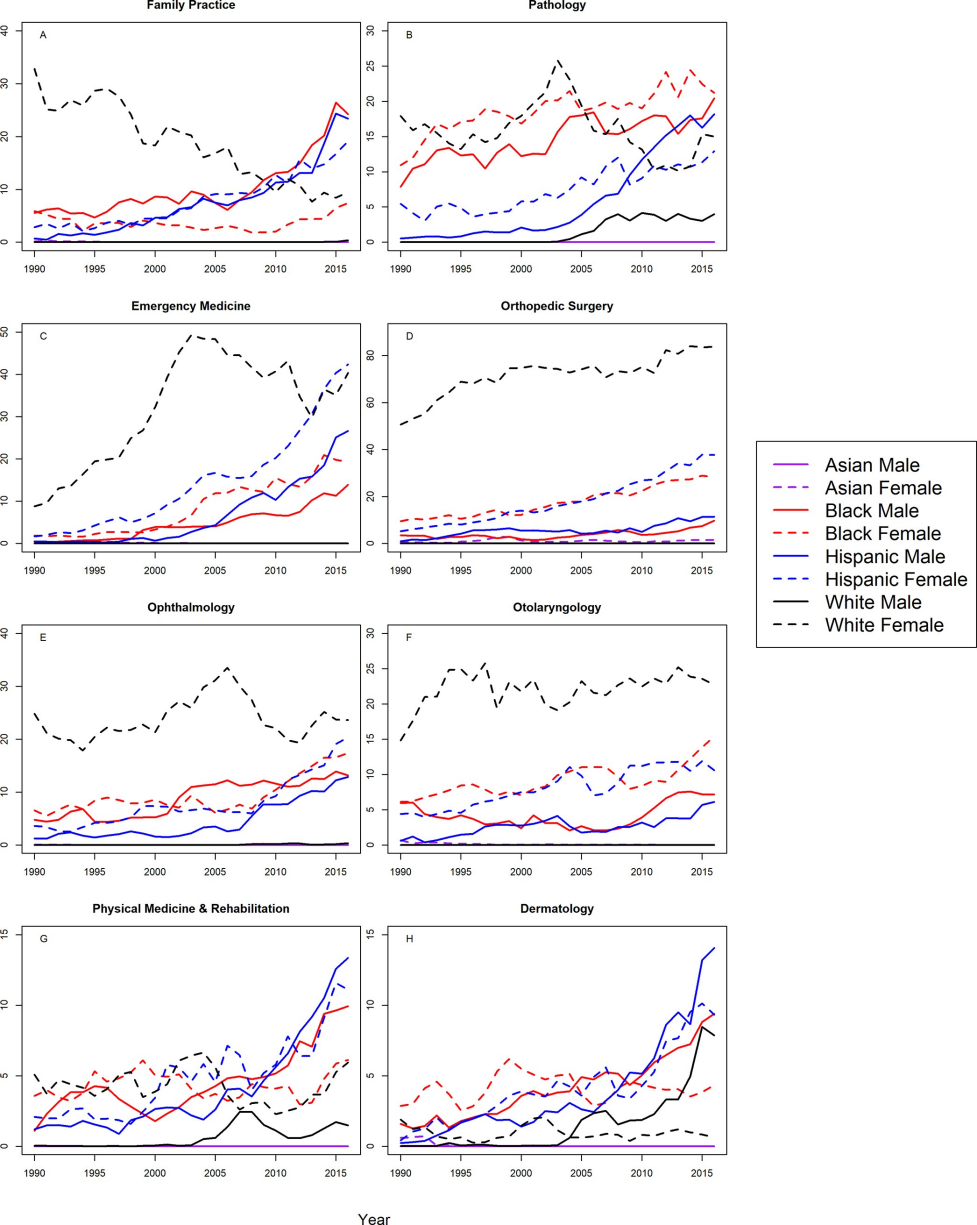
S-score for assistant professors by sex, race/ethnicity and department (continued).

Figs 1 and 2 shows the S-scores by year for assistant professors in 16 specialties for all groups. Correlation coefficients between year and S-score with 95% CIs are shown in Table 2 for assistant professors in all 16 specialties. At the assistant professor level, Hispanic males and females were underrepresented in all 16 specialties with trends toward greater underrepresentation with statistically significant, positive correlation coefficients. Similarly, Blacks were more underrepresented in 2016 in 15 of the 16 specialties analyzed, with one notable exception; Black females in OB/GYN were represented on par with the Census by 2016. For the remaining specialties, there were significant trends towards greater underrepresentation for Black males and females in in internal medicine, pediatrics, surgery, psychiatry, radiology, anesthesiology, neurology, emergency medicine, orthopedic surgery, and ophthalmology, Black males in OB/GYN, family practice, and PMR, and black females in otolaryngology. There were no significant trends for Black males in otolaryngology, or Black females in family practice, PMR, or dermatology.

**Table 2:** Pearson correlation coefficients between S-score and year with 95% confidence intervals for assistant professors, 1990–2016.

Department	Asian		Black		Hispanic		White	
	Male	Female	Male	Female	Male	Female	Male	Female
Internal Medicine	--	-0.37 (-0.65, 0.02)	0.95 (0.88, 0.98)	0.91 (0.81, 0.96)	0.85 (0.7, 0.93)	0.94 (0.87, 0.97)	0.62 (0.31, 0.81)	0.55 (0.22, 0.77)
Pediatrics	--	--	0.9 (0.79, 0.95)	0.92 (0.84, 0.97)	0.9 (0.79, 0.95)	0.86 (0.71, 0.93)	0.87 (0.73, 0.94)	-0.9 (-0.95, -0.79)
Surgery	--	-0.74 (-0.87, -0.5)	0.93 (0.85, 0.97)	0.97 (0.93, 0.98)	0.81 (0.62, 0.91)	0.96 (0.91, 0.98)	--	0.76 (0.54, 0.89)
Psychiatry	-0.3 (-0.61, 0.09)	-0.4 (-0.68, -0.02)	0.98 (0.96, 0.99)	0.5 (0.15, 0.74)	0.93 (0.84, 0.97)	0.84 (0.68, 0.92)	0.62 (0.31, 0.81)	-0.93 (-0.97, -0.86)
Radiology	--	-0.42 (-0.69, -0.04)	0.97 (0.94, 0.99)	0.9 (0.79, 0.95)	0.93 (0.85, 0.97)	0.9 (0.8, 0.96)	0.26 (-0.13, 0.58)	0.92 (0.83, 0.96)
Anesthesiology	--	-0.31 (-0.62, 0.08)	0.86 (0.71, 0.93)	0.86 (0.72, 0.94)	0.8 (0.6, 0.9)	0.93 (0.86, 0.97)	0.36 (-0.02, 0.65)	-0.48 (-0.73, -0.12)
Obstetrics & Gynecology	0.46 (0.1, 0.71)	-0.47 (-0.72, -0.11)	0.85 (0.69, 0.93)	-0.91 (-0.96, -0.82)	0.94 (0.87, 0.97)	0.84 (0.67, 0.92)	0.87 (0.74, 0.94)	-0.84 (-0.92, -0.67)
Neurology	-0.33 (-0.63, 0.06)	-0.23 (-0.56, 0.17)	0.97 (0.93, 0.99)	0.97 (0.94, 0.99)	0.88 (0.75, 0.94)	0.91 (0.81, 0.96)	0.66 (0.37, 0.83)	-0.17 (-0.51, 0.23)
Family Practice	-0.68 (-0.84, -0.4)	-0.71 (-0.86, -0.46)	0.83 (0.66, 0.92)	0.06 (-0.33, 0.43)	0.92 (0.83, 0.96)	0.95 (0.89, 0.98)	0.49 (0.14, 0.74)	-0.95 (-0.98, -0.89)
Pathology	--	-0.29 (-0.6, 0.1)	0.86 (0.72, 0.94)	0.86 (0.71, 0.93)	0.91 (0.81, 0.96)	0.91 (0.82, 0.96)	0.87 (0.74, 0.94)	-0.3 (-0.61, 0.09)
Emergency Medicine	-0.58 (-0.79, -0.25)	-0.58 (-0.79, -0.26)	0.95 (0.89, 0.98)	0.95 (0.9, 0.98)	0.9 (0.79, 0.95)	0.94 (0.87, 0.97)	-0.34 (-0.64, 0.05)	0.74 (0.49, 0.87)
Orthopedic Surgery	-0.32 (-0.63, 0.07)	0.2 (-0.2, 0.54)	0.71 (0.45, 0.86)	0.97 (0.94, 0.99)	0.85 (0.68, 0.93)	0.97 (0.95, 0.99)	--	0.87 (0.74, 0.94)
Ophthalmology	-0.33 (-0.63, 0.06)	-0.6 (-0.8, -0.28)	0.91 (0.81, 0.96)	0.77 (0.54, 0.89)	0.87 (0.74, 0.94)	0.87 (0.74, 0.94)	0.75 (0.52, 0.88)	0.27 (-0.12, 0.59)
Otolaryngology	-0.4 (-0.68, -0.02)	-0.73 (-0.87, -0.48)	0.33 (-0.06, 0.63)	0.79 (0.59, 0.9)	0.78 (0.58, 0.9)	0.92 (0.84, 0.96)	0.47 (0.11, 0.72)	0.41 (0.04, 0.68)
Physical Med. & Rehab.	0.32 (-0.07, 0.63)	0.16 (-0.23, 0.51)	0.83 (0.65, 0.92)	0.12 (-0.28, 0.47)	0.86 (0.71, 0.94)	0.88 (0.76, 0.95)	0.72 (0.47, 0.86)	-0.18 (-0.53, 0.21)
Dermatology	-0.27 (-0.59, 0.12)	-0.59 (-0.79, -0.27)	0.96 (0.91, 0.98)	0.11 (-0.28, 0.47)	0.87 (0.73, 0.94)	0.91 (0.8, 0.96)	0.81 (0.62, 0.91)	-0.16 (-0.51, 0.24)

--indicates groups for which the correlation coefficient was not estimable because the S-score was zero throughout the study period (no variance or covariance)

White females were underrepresented in 2016 in internal medicine, surgery, radiology, emergency medicine orthopedic surgery, and otolaryngology with significant trends toward decreased representation. White females were also underrepresented in neurology, pathology, ophthalmology, and PMR, with no significant trend. White females were underrepresented in anesthesiology and psychiatry with significant trends toward increased representation. White females transitioned from underrepresented to representation on par with the Census in pediatrics and psychiatry, and overrepresentation in OB/GYN, with significant trends toward representation on par with the Census. White females were represented on par with the Census in dermatology throughout the study period.

White males were overrepresented in the assistant professorship for surgery, radiology, anesthesiology, emergency medicine, orthopedic surgery, and otolaryngology for all years. White males trended from overrepresentation to representation on par with the Census in family practice, ophthalmology, and PMR. White males trended from overrepresentation in 1990, to underrepresentation in 2016 in internal medicine (S-score = 2.618), and this trend was statistically significant (r = 0.62, 95% CI: 0.31 to 0.81). However, as shown in Fig 1, the S-score for White males in 2016 was substantially lower than S-scores for Hispanics, Blacks, and White females (S-scores>70), suggesting that underrepresentation for White males among assistant professors in internal medicine, was less severe than underrepresentation for Hispanics, Blacks, and White females. This was also true in neurology, psychiatry, pathology, and dermatology where White males were underrepresented in 2016; but to lesser degree than Hispanics, Blacks, and White females in the same specialties. White males were underrepresented in pediatrics and OB/GYN with a significant trend toward greater underrepresentation.

Asian females were overrepresented in the assistant professorship from 1990–2016 in internal medicine, pediatrics, psychiatry, radiology, anesthesiology, OB/GYN, pathology and PMR. Asian females became overrepresented by 2008 or earlier, in surgery, neurology, family practice, emergency medicine, ophthalmology, otolaryngology, and dermatology. Asian females had representation on par with the Census (S-score between 0.0109 and 1.602) in orthopedic surgery throughout the study period. Asian males were overrepresented (S-score <0.0109) for all years (1990–2016) in the assistant professorship for all specialties except family practice, for which they became overrepresented from 1991 onward.

### Associate professors

**Fig 3:**
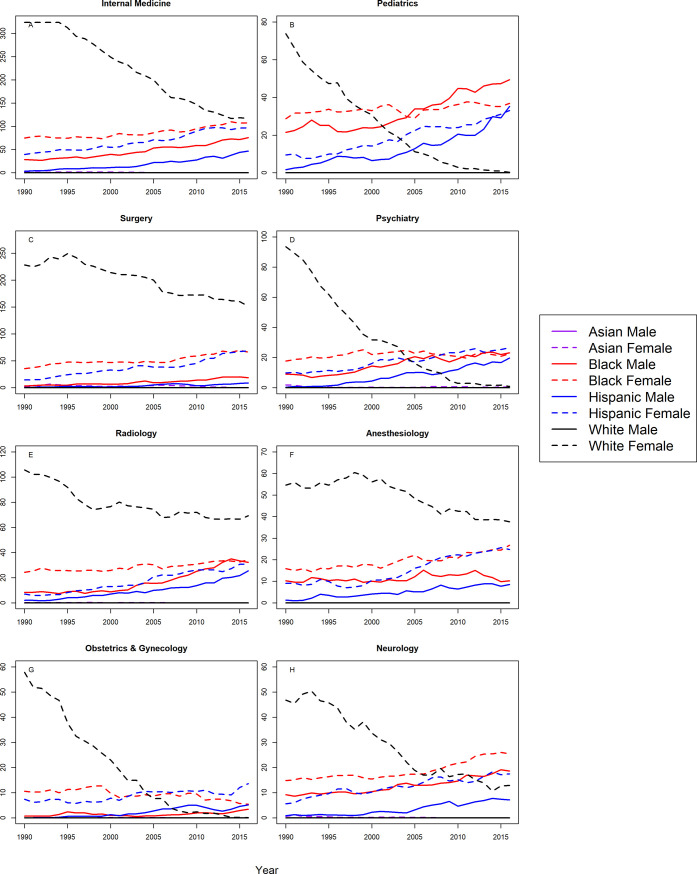
S-score for associate professors by sex, race/ethnicity and department.

**Fig 4:**
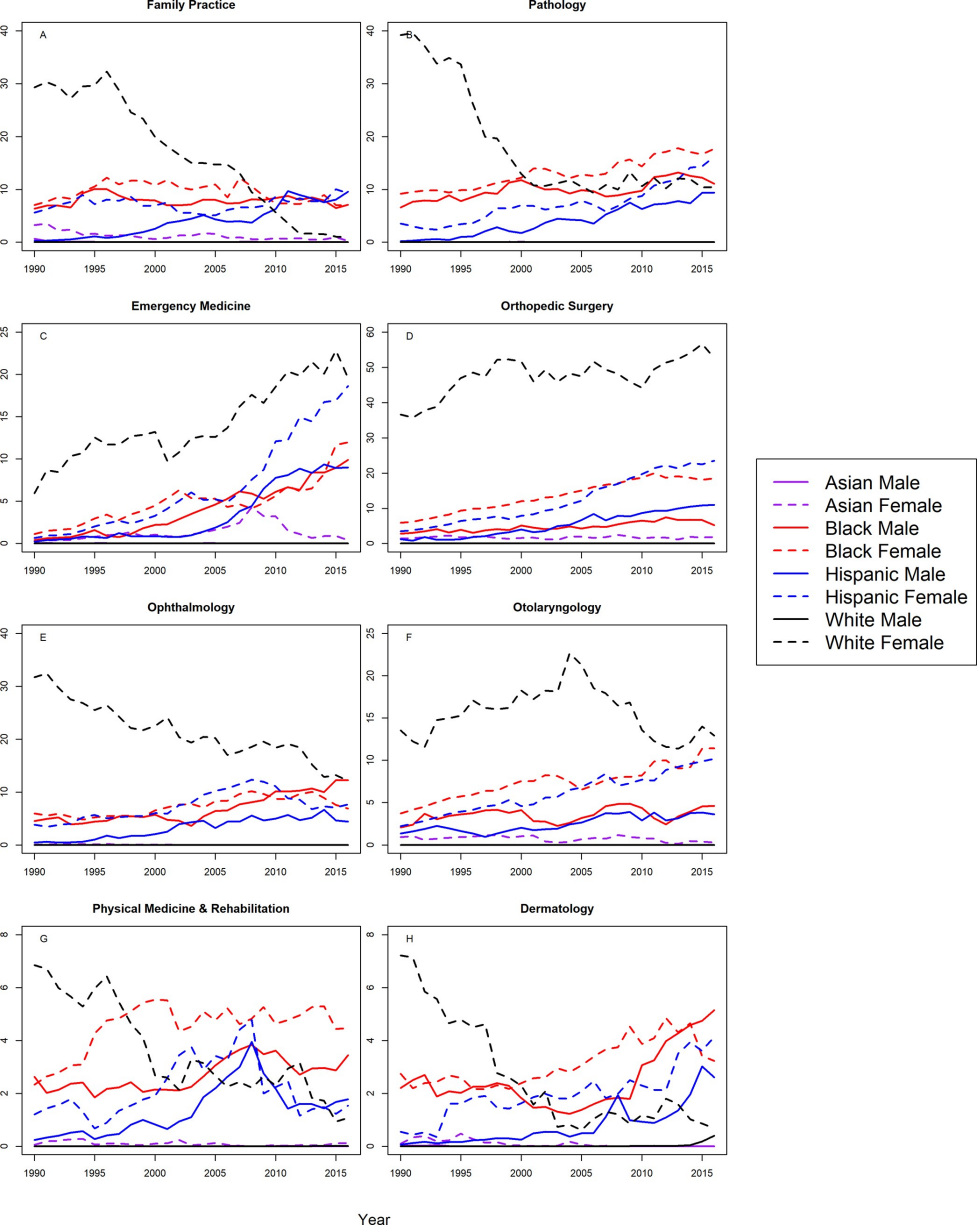
S-score for associate professors by sex, race/ethnicity and department (continued).

Figs 3 and 4 show trends in representation amongst Associate Professors for the 16 specialties, and Table 3 shows the correlation coefficients and CIs. At the associate professor level, Blacks and Hispanics were more underrepresented in 2016 than in 1990 for both sexes and in all 16 specialties with one exception; Black female associate professors in OB/GYN were less underrepresented in 2016 than 1990. Furthermore, there was a significant trend toward greater underrepresentation for Blacks and Hispanics of both sexes in all specialties with the following exceptions: Black females in OB/GYN exhibited a significant trend toward representation on par with the Census; Blacks in family practice and Black females in psychiatry exhibited no significant trend, and Hispanic females in Family Practice and PMR exhibited no significant trends.

**Table 3:** Pearson correlation coefficients between S-score and year with 95% confidence intervals for associate professors, 1990–2016.

Department	Asian		Black		Hispanic		White	
	Male	Female	Male	Female	Male	Female	Female	Male
Internal Medicine	--	-0.85 (-0.93, -0.69)	0.98 (0.95, 0.99)	0.92 (0.84, 0.96)	0.96 (0.92, 0.98)	0.98 (0.95, 0.99)	--	-0.99 (-1, -0.98)
Pediatrics	--	-0.18 (-0.52, 0.22)	0.93 (0.85, 0.97)	0.67 (0.39, 0.84)	0.94 (0.86, 0.97)	0.98 (0.95, 0.99)	0.35 (-0.04, 0.64)	-0.97 (-0.98, -0.93)
Surgery	--	-0.66 (-0.83, -0.38)	0.95 (0.89, 0.98)	0.92 (0.83, 0.96)	0.88 (0.76, 0.95)	0.97 (0.93, 0.98)	--	-0.95 (-0.98, -0.89)
Psychiatry	0.03 (-0.35, 0.41)	-0.34 (-0.64, 0.05)	0.95 (0.9, 0.98)	0.35 (-0.03, 0.65)	0.98 (0.96, 0.99)	0.97 (0.94, 0.99)	--	-0.96 (-0.98, -0.91)
Radiology	--	-0.28 (-0.6, 0.11)	0.93 (0.85, 0.97)	0.9 (0.8, 0.96)	0.96 (0.92, 0.98)	0.98 (0.96, 0.99)	--	-0.89 (-0.95, -0.77)
Anesthesiology	0 (-0.38, 0.38)	0.57 (0.25, 0.78)	0.49 (0.14, 0.74)	0.94 (0.88, 0.97)	0.96 (0.91, 0.98)	0.94 (0.86, 0.97)	--	-0.88 (-0.94, -0.75)
Obstetrics & Gynecology	0.42 (0.05, 0.69)	0.04 (-0.35, 0.41)	0.55 (0.22, 0.77)	-0.79 (-0.9, -0.58)	0.92 (0.82, 0.96)	0.85 (0.69, 0.93)	0.51 (0.17, 0.75)	-0.96 (-0.98, -0.9)
Neurology	-0.16 (-0.51, 0.23)	-0.79 (-0.9, -0.59)	0.96 (0.9, 0.98)	0.9 (0.79, 0.95)	0.94 (0.87, 0.97)	0.95 (0.9, 0.98)	0.33 (-0.06, 0.63)	-0.97 (-0.99, -0.93)
Family Practice	-0.47 (-0.72, -0.11)	-0.77 (-0.89, -0.55)	-0.03 (-0.4, 0.36)	-0.31 (-0.62, 0.08)	0.95 (0.88, 0.98)	0.3 (-0.09, 0.61)	0.35 (-0.04, 0.64)	-0.98 (-0.99, -0.95)
Pathology	--	-0.16 (-0.51, 0.23)	0.76 (0.53, 0.88)	0.96 (0.9, 0.98)	0.97 (0.94, 0.99)	0.92 (0.84, 0.97)	0.47 (0.11, 0.72)	-0.84 (-0.92, -0.67)
Emergency Medicine	-0.67 (-0.84, -0.38)	0.4 (0.02, 0.67)	0.97 (0.93, 0.99)	0.87 (0.74, 0.94)	0.9 (0.79, 0.95)	0.94 (0.86, 0.97)	-0.33 (-0.63, 0.06)	0.93 (0.85, 0.97)
Orthopedic Surgery	-0.48 (-0.73, -0.13)	0.03 (-0.35, 0.41)	0.89 (0.77, 0.95)	0.98 (0.96, 0.99)	0.98 (0.95, 0.99)	0.98 (0.95, 0.99)	--	0.72 (0.47, 0.86)
Ophthalmology	-0.53 (-0.76, -0.19)	-0.74 (-0.88, -0.51)	0.89 (0.77, 0.95)	0.8 (0.6, 0.9)	0.93 (0.84, 0.97)	0.69 (0.43, 0.85)	0.46 (0.09, 0.71)	-0.95 (-0.98, -0.89)
Otolaryngology	-0.57 (-0.78, -0.24)	-0.54 (-0.77, -0.21)	0.39 (0.01, 0.67)	0.93 (0.86, 0.97)	0.85 (0.69, 0.93)	0.98 (0.96, 0.99)	0.34 (-0.05, 0.64)	-0.11 (-0.47, 0.28)
Physical Med. & Rehab.	0.21 (-0.19, 0.54)	-0.52 (-0.75, -0.17)	0.71 (0.44, 0.86)	0.61 (0.3, 0.8)	0.69 (0.42, 0.85)	0.25 (-0.15, 0.57)	0.52 (0.17, 0.75)	-0.91 (-0.96, -0.8)
Dermatology	-0.57 (-0.78, -0.24)	-0.73 (-0.87, -0.48)	0.58 (0.25, 0.78)	0.81 (0.62, 0.91)	0.83 (0.66, 0.92)	0.88 (0.75, 0.94)	0.49 (0.13, 0.73)	-0.87 (-0.94, -0.72)

-- indicates groups for which the correlation coefficient was not estimable because the S-score was zero throughout the study period (no variance or covariance)

White females were underrepresented in 2016 in internal medicine, surgery, radiology, anesthesiology, neurology, pathology, and ophthalmology, and there was a significant trend toward representation on par with the Census. White females were also underrepresented in emergency medicine and orthopedic surgery, and there was a significant trend towards greater underrepresentation for White females in these specialties. White females were underrepresented in otolaryngology where there was no statistically significant trend. White females trended from underrepresented to representation on par with the Census in pediatrics, psychiatry, OB/GYN, family practice, dermatology and PMR.

Asian females were overrepresented in 2016 and throughout most of the study period in internal medicine, pediatrics, radiology, anesthesiology, OB/GYN, neurology, pathology, and dermatology throughout the study period. Asian females were represented on par with the Census in surgery, psychiatry, family practice, emergency medicine, orthopedic surgery, otolaryngology, and PMR. Asian males were overrepresented in all specialties except family practice, where they were represented on par with the Census. White males were overrepresented in all specialties.

### Full professors

**Fig 5:**
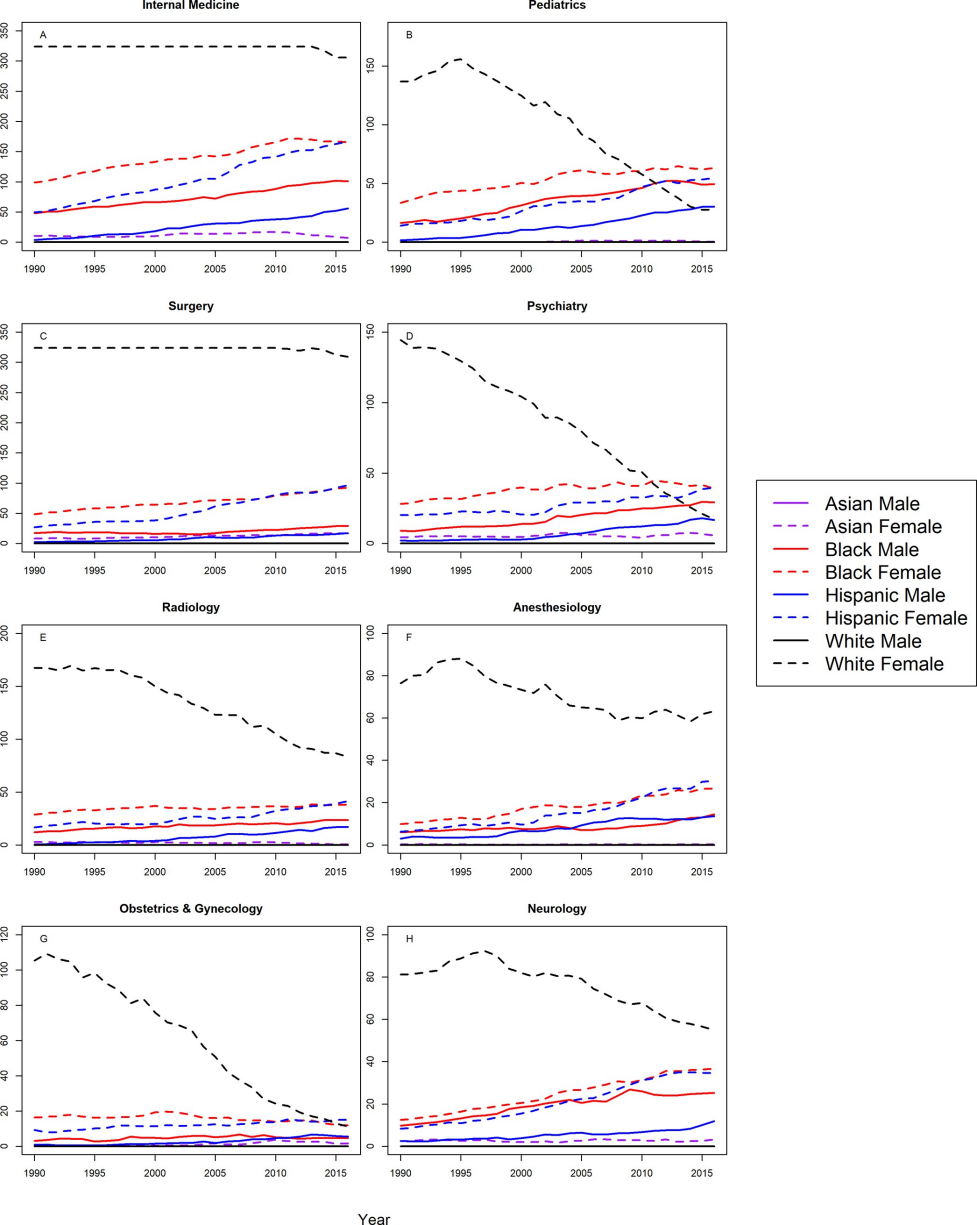
S-score for full professors by sex, race/ethnicity and department.

**Fig 6:**
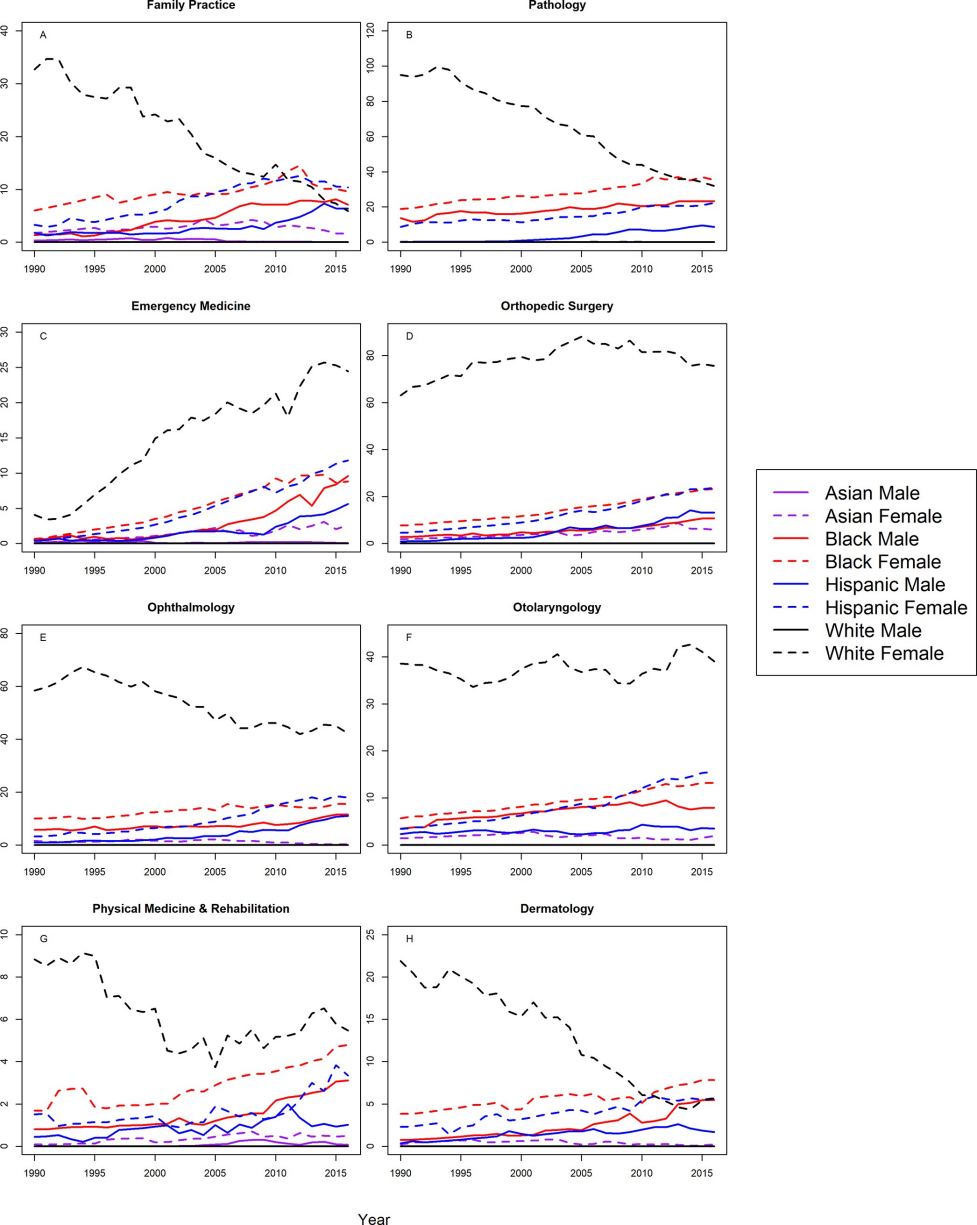
S-score for full professors by sex, race/ethnicity and department (continued).

Figs 5 and 6 show S-scores by year for the 16 specialties, and Table 4 shows correlation coefficients and CIs for full professors. Blacks and Hispanics were more underrepresented in 2016 than in 1990 for all specialties, and there were significant trends towards greater underrepresentation with one exception; Black female full professors in OB/GYN exhibited a significant trend toward representation on par with the Census.

**Table 4:** Pearson correlation coefficients between S-score and year with 95% confidence intervals for full professors, 1990–2016.

Department	Asian		Black		Hispanic		White	
	Male	Female	Male	Female	Male	Female	Male	Female
Internal Medicine	--	0.39 (0.02, 0.67)	0.99 (0.98, 1)	0.98 (0.96, 0.99)	0.99 (0.98, 1)	0.99 (0.99, 1)	--	-0.52 (-0.75, -0.17)
Pediatrics	-0.08 (-0.45, 0.31)	0.75 (0.51, 0.88)	0.98 (0.96, 0.99)	0.96 (0.91, 0.98)	0.99 (0.97, 0.99)	0.98 (0.97, 0.99)	--	-0.96 (-0.98, -0.92)
Surgery	-0.07 (-0.44, 0.32)	0.96 (0.92, 0.98)	0.78 (0.56, 0.89)	0.99 (0.98, 1)	0.98 (0.96, 0.99)	0.98 (0.95, 0.99)	--	-0.58 (-0.79, -0.26)
Psychiatry	0.12 (-0.27, 0.48)	0.5 (0.15, 0.74)	0.99 (0.97, 0.99)	0.87 (0.73, 0.94)	0.95 (0.9, 0.98)	0.94 (0.87, 0.97)	--	-1 (-1, -0.99)
Radiology	--	-0.71 (-0.86, -0.45)	0.97 (0.93, 0.99)	0.87 (0.72, 0.94)	0.98 (0.96, 0.99)	0.94 (0.87, 0.97)	--	-0.98 (-0.99, -0.96)
Anesthesiology	0.22 (-0.18, 0.55)	-0.15 (-0.5, 0.25)	0.83 (0.67, 0.92)	0.99 (0.97, 0.99)	0.96 (0.92, 0.98)	0.97 (0.93, 0.98)	--	-0.89 (-0.95, -0.78)
Obstetrics & Gynecology	0.47 (0.12, 0.72)	0.78 (0.56, 0.89)	0.53 (0.18, 0.76)	-0.71 (-0.86, -0.45)	0.94 (0.87, 0.97)	0.95 (0.89, 0.98)	--	-0.99 (-1, -0.98)
Neurology	0.05 (-0.34, 0.42)	0.08 (-0.31, 0.45)	0.96 (0.91, 0.98)	1 (0.99, 1)	0.94 (0.88, 0.97)	0.99 (0.97, 0.99)	--	-0.89 (-0.95, -0.76)
Family Practice	-0.66 (-0.83, -0.37)	0.25 (-0.14, 0.58)	0.97 (0.93, 0.99)	0.8 (0.6, 0.9)	0.85 (0.69, 0.93)	0.95 (0.88, 0.97)	--	-0.98 (-0.99, -0.96)
Pathology	--	0.08 (-0.31, 0.45)	0.94 (0.88, 0.97)	0.98 (0.95, 0.99)	0.95 (0.89, 0.98)	0.96 (0.91, 0.98)	--	-0.99 (-0.99, -0.97)
Emergency Medicine	-0.37 (-0.66, 0.02)	0.92 (0.82, 0.96)	0.89 (0.78, 0.95)	0.98 (0.96, 0.99)	0.88 (0.76, 0.95)	0.98 (0.96, 0.99)	-0.33 (-0.63, 0.06)	0.97 (0.94, 0.99)
Orthopedic Surgery	0.11 (-0.28, 0.47)	0.93 (0.84, 0.97)	0.95 (0.9, 0.98)	0.99 (0.98, 1)	0.95 (0.89, 0.98)	0.98 (0.96, 0.99)	--	0.63 (0.33, 0.82)
Ophthalmology	-0.24 (-0.57, 0.16)	-0.52 (-0.75, -0.17)	0.88 (0.74, 0.94)	0.93 (0.86, 0.97)	0.92 (0.84, 0.96)	0.97 (0.92, 0.98)	--	-0.92 (-0.96, -0.83)
Otolaryngology	-0.56 (-0.77, -0.22)	-0.34 (-0.64, 0.04)	0.89 (0.76, 0.95)	0.99 (0.98, 1)	0.64 (0.35, 0.82)	0.97 (0.94, 0.99)	--	0.36 (-0.02, 0.65)
Physical Med. & Rehab.	0.67 (0.39, 0.84)	0.81 (0.62, 0.91)	0.9 (0.79, 0.95)	0.89 (0.77, 0.95)	0.72 (0.47, 0.87)	0.71 (0.44, 0.86)	-0.07 (-0.44, 0.32)	-0.7 (-0.85, -0.43)
Dermatology	-0.38 (-0.67, 0)	-0.59 (-0.79, -0.27)	0.92 (0.84, 0.97)	0.91 (0.81, 0.96)	0.9 (0.78, 0.95)	0.94 (0.88, 0.97)	0.47 (0.1, 0.72)	-0.97 (-0.99, -0.94)

-- indicates groups for which the correlation coefficient was not estimable because the S-score was zero throughout the study periods (no variance or covariance)

White females were underrepresented in internal medicine, pediatrics, surgery, psychiatry, radiology, anesthesiology, OB/GYN, neurology, family practice, pathology, ophthalmology, PMR, and dermatology but there were significant trends towards representation on par with the Census in these specialties. White females were also underrepresented in emergency medicine and orthopedic surgery; however, the trends were towards greater underrepresentation. White females were underrepresented in otolaryngology where there was no statistically significant trend.

Asian females were underrepresented in internal medicine and neurology where the trend was toward representation on par with the Census. Asian females were underrepresented in surgery, orthopedic surgery and emergency medicine, where the trends were toward greater underrepresentation. Asian females were underrepresented in psychiatry where there was a trend toward greater representation on par with the Census, but it was not statistically significant. Asian females were represented on par with the Census in pediatrics, radiology, anesthesiology, OB/GYN, family practice, ophthalmology, otolaryngology, PMR, and dermatology. Asian females were overrepresented among full professors in pathology.

Asian males were overrepresented in all specialties except emergency medicine and PMR where they were represented on par with the Census. White males were overrepresented in all specialties at the full professor level.

## Discussion

This study demonstrates that Blacks and Hispanics were more underrepresented in 2016 than in 1990 at the assistant, associate, and full professor level among US clinical medical faculty in nearly all specialties. Further, in most specialties, there were significant trends toward greater underrepresentation. One notable exception was in OB/GYN where black females were represented on par with the Census at the assistant professor level and were trending towards greater representation at the associate and full professor levels. White females were also underrepresented in many specialties, particularly at the associate and full professor levels, but generally trended toward representation on par with the Census.

Many previous studies have investigated trends in diversity across different medical specialties and at different levels of training, including surgery[7], obstetrics and gynecology [8], radiation oncology[12], diagnostic radiology[11], dermatology [9,13], physical medicine and rehabilitation[10], and ophthalmology[15] as well as graduate medical education[23] and academic faculty[14] overall. However, these studies only assessed raw number and proportions of medical faculty, and while many of them noted URM racial groups and their corresponding proportion in the overall population, none statistically quantified deviation from demographics of the US, or the degree of underrepresentation. This approach can lead to erroneous conclusions that do not adequately capture how well the composition of the physician workforce mimics the US. For example, among academic faculty overall, Guevara *et al*. noted a modest overall increase URM faculty in the US from 6.8% to 8.0% between 2000 and 2010 [14]. However, in the same time span the Hispanic population in the US grew from 12.5% to 16.3%, and the Black population grew from 12.3% to 12.6%; an overall 4.1% (24.8% to 28.9%) increase in the proportion of Blacks and Hispanics in the US. Therefore, the 1.2% gain noted by Guevara *et al*., actually corresponds to a widening gap in faculty representation of URM populations in the US. Our new metric, the S-score, captures the changes in the US population and accounts for this as an increase in underrepresentation. We demonstrate this in our examination of numerous specialties, where changes in the S-score show increased underrepresentation despite reported increased raw counts and proportions of Hispanic and Black faculty. The S-score detects if the representation of URM groups in academic URM faculty exceeds, is commensurate with, or is deficient relative to the population distribution and corresponds to a statistical test of the AAMC’s definition of URM in medicine.

We created the S-score to be used by the major governing bodies of the fields of medicine (e.g. the American Board of Radiology (ABR) and the American Association of Orthopedic Surgeons (AAOS)). The goal was that these bodies could use the S-score to assess progress in increasing diversity in their respective fields at the national level. The S-score is a statistical test, and much like a t-test can be used to assess change in diversity not just among academic physicians over time, but also medical residents and non-academic physicians. The S-score not only accurately and objectively measures underrepresentation in medicine, but it can be used to assess future progress in achieving diversity in other fields.

The S-score can also be tailored to a particular geography, even those of different countries, if Census data is available. For example, the S-score for any university/school can take into consideration the local racial/ethnic demographic distribution, as opposed to the broader national demographics. The challenge here is, how does one define the local geography of a university/academic center. Another challenge is that even though the local geography is defined, the accompanying census demographics for that geography may be unknown. Furthermore, because racial/ethnic demographic distributions vary by local geography, two institutions with the same ethnic/racial make-up may have widely different S-scores, however, if each academic center reflects the local racial/ethnic demographic distributions, then nationally, there will be no difference in representation.

URM underrepresentation among faculty is driven by multiple factors including racial and ethnic biases in hiring, promotion[24], and compensation[25] that may limit URM faculty recruitment and retention. Addressing this underrepresentation is an imperative in medicine. In the US there are significant racial and ethnic health disparities; however, most clinical practice is based on studies that were conducted primarily in white male populations[26]. Therefore, the proportion of the US population that are not addressed by these studies is rising as the US becomes more diverse. URM involvement in research enhances minority study recruitment and can help increase participation from traditionally hard to reach populations. Also, diverse institutions train physicians that more effectively serve minority communities so by improving representation we can indirectly improve minority health [27]. Given the long duration of medical training and the time between faculty promotions, it is important that interventions to increase diversity are applied at every step along the pipeline from medical student to professor, so that we can reverse these troubling trends. We must also be cautious in considering fields where traditional majority demographics, like white males, are underrepresented because this may be driven by factors related to increased access to educational and professional resources allowing them to select against certain specialties.

This study has a few limitations. Firstly, the AAMC Faculty Roster is voluntarily reported and may not be inclusive of all medical faculty in allopathic medical schools, however, this Faculty Roster comprises the majority of clinical medical faculty in the US, and we suspect that any missingness is likely completely random and therefore would not bias our results[17]. However, our results do not include osteopathic medical schools which may exhibit different trends in racial and ethnic representation. Also, categorizations of race and ethnicity in both the US Census and the Faculty Roster are limited. The Faculty Roster grouped Hispanic, Latino, and Spanish origin, as a single category, and similarly included only a single category to represent all individuals of Asian descent. However, the diversity within both of these populations are well documented so it is possible that there is variability in representation within this group that our study was unable to capture. Similarly, the US Census is flawed and noted by policy makers to be subject to multiple forms of sampling bias[28] which may further limit the true accuracy of the S-score.

Another limitation of this study is that the S-score is based on US Census proportions; therefore, the racial/ethnic definitions of the US Census must be used for faculty data collection if S-scores are to be calculated. However, there exist several important underrepresented groups, specifically gender and sexual minority groups that are not captured in this analysis and not reported as part of the Census or Faculty Roster. As medicine and society move toward a better understanding of the nuances of gender and sexual diversity, it is important to ensure that the medical workforce reflects the population of the communities they serve and improves representation of non-cisgender, non-heterosexual clinicians. This is of great importance when we consider the documented health disparities that face some of these populations, notably transgender women of color, and the potentially unknown barriers to care that these communities may face[29,30].

One might question how representative the work force in academic medicine is of the overall medical community. For example, non-academic medical positions tend to be more lucrative, thus inclusion of these positions in the analysis may more adequately reflect the population. There is data to suggest that the workforce in academic medicine is less diverse than the workforce in non-academic medicine[11]. However, there are also several articles showing that URM physicians are more likely to work in underserved areas and make less than their non-URM physician colleagues[1–5,25], which suggests that URM physicians may not be as financially motivated as non-URM physician colleagues. If we assume that neither being an academic physician nor being a non-academic physician is associated with race (i.e. the decision to choose one or the other is independent of both race), the racial make-up of academic medicine should reflect that seen in the US. The fact that the academic physician workforce appears different from the US Census may reflect strong environmental pressure that reduces the number of URM physicians in academia. The question of why these differences exist needs to be answered and we think the S-score will be a powerful tool during this process.

It is also important to discuss the age of the population included in this study. For the purposes of our analysis, we restricted population estimates to only include all individuals the age of 30 to 84 to represent the subset of the population eligible to be clinical faculty. This was to account for the different age distribution between minorities and whites in the US[20]. One limitation is that this age group may not accurately represent the extremes of ages among faculty; there may exist faculty younger than 30 or older than 84, and the age distribution of the physicians in academia may vary over time. Further, the promotion process is highly variable within and between institutions. Some physicians may stay their entire careers as instructors, others may skip levels and be hired as associate professors or full professors. There are no defined age cutoffs for these groups, so it makes the analysis difficult to perform without making assumptions about the ages of each professorial group, thereby introducing error into the analysis. However, this limitation may actually underestimate the discrepancy between the racial and ethnic composition of the population of patients, and the physician workforce. A similar analysis performed by the authors including the US population of all ages, showed steeper trends toward declining representation among many specialties, therefore, any differences in representation cannot be explained by the relatively younger age of the URM population. Lastly, previously published data show that the URM pediatric population is significantly more likely to be cared for by URM physicians[31,32] and that the URM pediatric population is subject to the harmful effects of implicit bias in the clinical setting[33]. Ignoring the need for more racial and ethnic diversity amongst pediatricians and other clinicians that take care of children could negatively impact the health of minority children.

Finally, it is important to note that rate-ratios work and will lead to similar conclusions/inference as those obtained from the S-score. However, they do not have the same accessibility as the S-score. For example, if one says the rate-ratio of Hispanics (the proportion of academic faculty that are Hispanic divided by the proportion of individuals in the US population that are Hispanic) decreased from 0.7 to 0.6, this says Hispanics are less well represented over time, but does not really state the urgency or real magnitude of the problem. The S-score is a simple probability–the probability that the proportion of Hispanic faculty seen are a random sample from the US population. If one reports the S-score increased from 45 to 50, then that is more easily accessible to most scientists and, perhaps more importantly to the layperson and policy makers who may influence decisions related to these findings. An S-score of 50 corresponds to a P-value of 10^−50^. The odds of winning the Powerball is 1/292,201,338. The odds of seeing the number of Hispanic faculty given an S-score of 50 is 1/10^50^. So, one can state, an S-score of 50 is similar to the odds of winning the Powerball approximately 6 times (i.e. an extremely unlikely random event).

## Conclusion

Increasing diversity in academic medicine is an important step toward reaching health equity for underserved communities[5]. Institutions with diverse faculty are better equipped to address the needs of marginalized populations through research and patient care, and are more suitable for training the next generation of physicians to practice culturally relevant medicine[27]. Our study shows that we have made limited strides toward this crucial goal, with Blacks and Hispanics being more underrepresented in clinical academic medicine compared to 27 years ago in 1990, despite the advent of initiatives, pipeline programs, training programs, minority faculty development programs and offices of diversity and inclusion[27,34–37]. Therefore, we advocate for more aggressive recruitment and retention programs, and elimination of documented biases in faculty promotion[24]. As we continue to improve, the S-score can provide a statistically objective measure of charting our progress towards creating a diverse physician workforce.

## Supporting information

S1 Appendix Justification for the S-score. (DOCX)

S1 Fig Observed and expected change in Hispanic faculty at a hypothetical academic institution. (TIFF)
