# Supplementary material for: Declining racial and ethnic representation in clinical academic medicine: A longitudinal study of 16 US medical specialties
Source: PLoS One. 2018 Nov 16;13(11):e0207274. doi: 10.1371/journal.pone.0207274 (PMC6239326; doi:10.1371/journal.pone.0207274)
Supplement: S1 Appendix — (DOCX) [file pone.0207274.s001.docx]

**S1 Appendix: Justification for the S-score**

A major obstacle in improving racial/ethnic and sex diversity is that there is no objective and accurate measure of diversity/representation. Whereas clinical trials, for example, have defined metrics such as overall survival or progression free survival, no appropriate metrics exist for diversity. Therefore, there is a need for a metric that can be used to assess progress in diversity/representation. Currently, changes in numbers or proportions of faculty are reported and used to assess progress, however, this appendix will show why these metrics can often result in erroneous conclusions using an example loosely based on real data. Consider the example case in S1 and S2 Tables and S1 Fig below:

S1 Table: Hispanic and Non-Hispanic faculty over time at a hypothetical academic institution, count, percent and S-score

| **Race/ethnicity** | **Year** | | |
| --- | --- | --- | --- |
|  | 2000 | 2014 | 2016 |
| Number of Hispanic faculty | 100 | 110 | 120 |
| Number of non-Hispanic faculty | 4900 | 4990 | 5380 |
| **Total faculty** | 5000 | 5100 | 5500 |
| % Hispanic faculty | 2.0% | 2.11% | 2.18% |
| % non-Hispanic faculty | 12.5% | 16.3% | 17.6% |
| **S-score for Hispanic faculty** | 162.91 | 243.09 | 293.35 |

S2 Table: Change in number of Hispanic faculty over time at a hypothetical academic institution

|  | 2000 | 2014 | 2016 |
| --- | --- | --- | --- |
| Change in number of Hispanic faculty from 2000 | - | 10% | 20% |
| Change in % of Hispanic faculty from 2000 | - | 5.5% | 9.0% |

**Changes in numbers**

S1 Table shows a hypothetical example of Hispanic faculty at an academic institution. When we evaluate the change in the numbers of Hispanic faculty at the institution, we find that Hispanic faculty have increased from 100 in 2000, to 120 in 2016 - a 20% increase! The conclusion is often made, based on this analysis, that there is increasing diversity amongst Hispanic faculty.

**Changes in proportions**

However, there is a concomitant increase in the number of non-Hispanic faculty from 2000 to 2016. When we evaluate changes in the proportions of faculty, we find that Hispanic faculty have increased from 2.0% of the total faculty in 2000 to 2.18% in 2016. This change appears smaller in magnitude than that observed when evaluating changes in numbers of faculty. Again, however, the conclusion often made is that there is an increase in the proportion of Hispanic faculty and thus an increase in Hispanic diversity.

**The S-score**

The Association of American Medical Colleges (AAMC) has defined under-represented in Medicine (URM) to mean those racial and ethnic populations that are under-represented in the medical profession relative to their numbers in the general population[16].

In the example, the number of Hispanic faculty increased by 20% between 2000 and 2016 and the percentage of Hispanic faculty increased from 2.0% in 2000 to 2.18% in 2016. However, the percentage of Hispanics in the general population (from US census data) increased from 12.5% in 2000 to 17.6% in 2016. According to the AAMC, under-representation is tied to a group’s representation in the US population, however neither the raw numbers of Hispanic faculty nor the proportion of Hispanic faculty take the change in the proportion of Hispanics in the US into consideration.

Based on the AAMC’s definition of under-representation, any metric used to determine whether a group is underrepresented should consider that group’s representation in the US population. The S-score does that.

We calculated the probability of finding the observed or a lower proportion of the group studied using the AAMC definition of underrepresentation in medicine. The null hypothesis was that the proportion of any group should equal the proportion of that same group in the US population based on census data.

S-scores are positive. S-scores greater than 1.602 (P-value < 0.025) indicate a group is under-represented and S-scores less than 0.011 (P-value> 0.975) indicate a group is over-represented. These thresholds were chosen so that only 5% of the population would be over- or under-represented, however, other thresholds are possible. The P-value is 10^-S-score^. The larger the S-score, the more underrepresented the group.

Based on this data, the S-score for Hispanic faculty increased from 162.91 in 2000 to 293.35 in

2016. While the number and proportion of Hispanic faculty increased, the S-score clearly shows that Hispanics are far more under-represented in 2016 than they were in 2000, contradicting the conclusions made from analyses of the number and proportion of Hispanic faculty.

Data on race/ethnicity must be collected in a similar fashion to that utilized by the US census in order to have comparable data for the S-score to be calculated/utilized. Therefore, data collection on race/ethnicity should simply follow the format utilized by the US census.

The S-score will be particularly helpful in determining the effectiveness of efforts used to support diversity over time. The S-score should also be used to determine where resources should be applied to support diversity. In the above example, based on previous metrics, it may be perceived that because the numbers and proportions of Hispanics are increasing, that resources should not be expended on that group, when in fact, Hispanic faculty need more support in 2016 to increase their representation in medicine because they are becoming even more under-represented.

Previous metrics used for diversity have been suboptimal in determining whether a group is under-represented according to the AAMC definition of under-representation in medicine. The

S-score is a statistically-based metric that solves this problem by taking into account the proportion of a group, the group’s representation in the US population, and the total number of individuals in all groups when determining whether a group is under or over-represented in medicine.

**[Insert S1 Fig]**

S1 Fig shows that the number of Hispanic faculty increase slowly from 2000 to 2016, however, the difference between the observed numbers (color coded vertical lines) from the expected histogram distribution based on a random sample from the US population is actually worsening.

The first step in solving any problem is to identify that problem – and we have identified that racial/ethnic and sex diversity in medicine is a problem. The second step in solving a problem is having a reliable, quantifiable metric that can be used to assess progress towards the goal of solving the problem of diversity in medicine – and the S-score is that metric. Public and private institutions, universities and departments should use the S-score to track annual progress towards achieving diversity.
