# Supplementary figures and images for: Declining racial and ethnic representation in clinical academic medicine: A longitudinal study of 16 US medical specialties
Source: PLoS One. 2018 Nov 16;13(11):e0207274. doi: 10.1371/journal.pone.0207274 (PMC6239326; doi:10.1371/journal.pone.0207274)

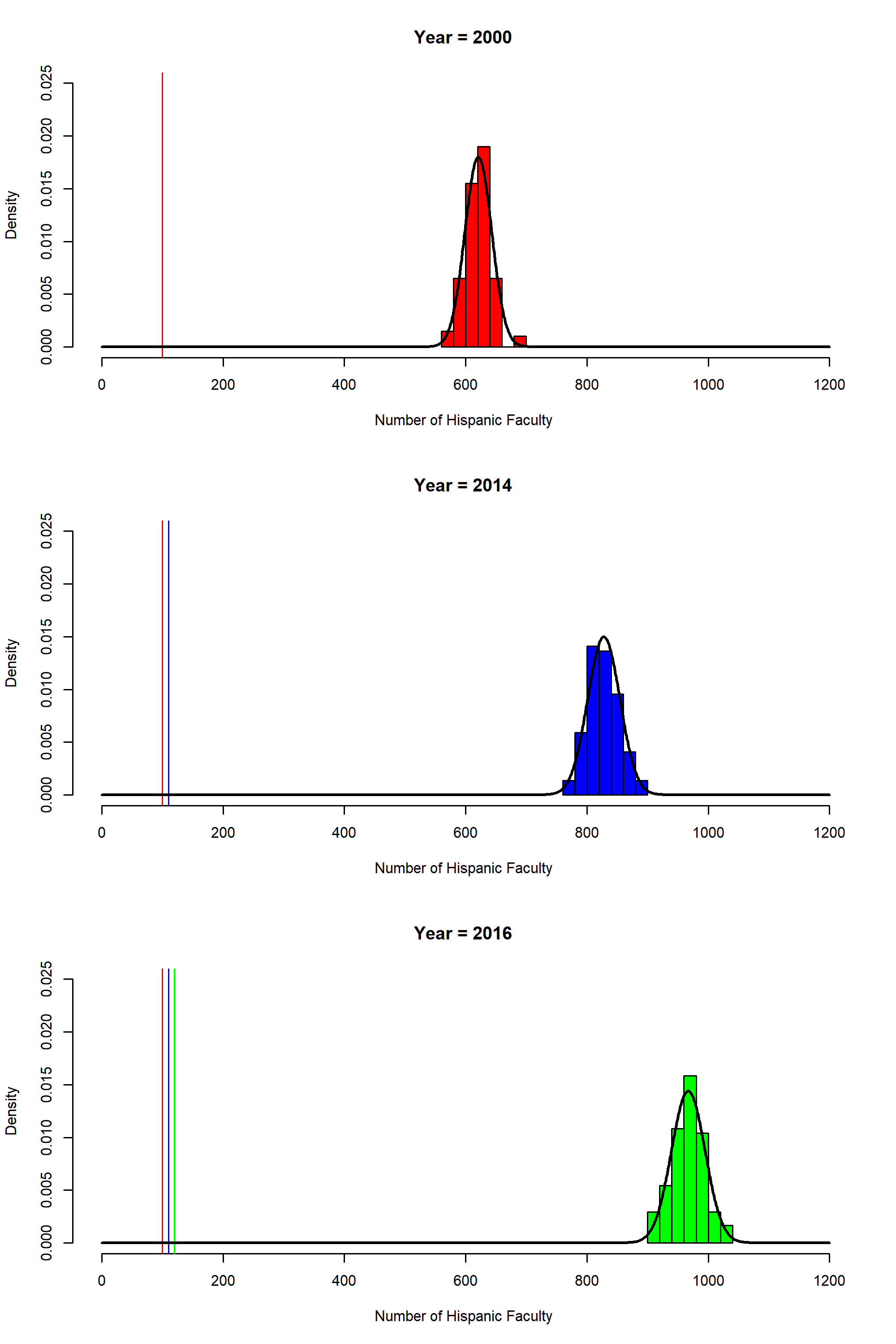

Supplement: S1 Fig — (TIFF) [file pone.0207274.s002.tiff]
